# A young doctor’s strange, adventurous journey from Turkey to Canada (with a few stops in between)

**DOI:** 10.1192/j.eurpsy.2026.10263

**Published:** 2026-07-31

**Authors:** S. Guloksuz

**Affiliations:** Department of Psychiatry, University of British Columbia, Vancouver, Canada

## Abstract

**Abstract:**

In this presentation, drawing on experiences from different health care systems and diverse academic environments around the globe, I will reflect on a journey across countries, institutions, and roles, and on the lessons learned while navigating uncertainty, competing clinical and academic demands, and a nonlinear career trajectory. I will discuss the value of adaptability as a deliberate process of recalibration rather than a sign of indecisiveness. Together with the audience, I will explore the following questions: How does one define a coherent professional identity amid constant transition? How can passion be pursued without being consumed by *passio*? Ultimately, I will attempt to argue that professional identity is not a fixed destination but an evolving construct.

**Disclosure of Interest:**

None Declared

